# Editorial: Nutrition and sustainable development goal 14: life below water

**DOI:** 10.3389/fnut.2024.1441616

**Published:** 2024-06-24

**Authors:** Miroslava R. Atanassova, Leila Ktari

**Affiliations:** ^1^Møreforsking AS, Norwegian Maritime Competence Center (NMK), Ålesund, Norway; ^2^National Institute of Marine Sciences and Technologies (INSTM), Carthage University, Tunis, Tunisia

**Keywords:** human, nutrition, health, sustainable use of resources, aquatic animals, diets, quality

Healthy and nutritious diets are of central importance to the United Nations (UN) Sustainable Development Goal (SDG) 14 “Life under water” targets, as basis for the improved conservation and sustainable use of oceans, seas and marine resources. The availability of abundant, nutritious sources is fundamental to high quality nutrition of both aquatic animals and humans and is intricately linked to the reduction of pollution and the effects of climate change. The role of aquatic foods in sustainable healthy diets was recently revised by UN's Nutrition section (1), as a lack of sufficient strategic promotion of their dietary effects was recognized. A policy brief with recommendations was emitted by UN on basis of the 2021 discussion article (2). Aquatic foods for human nutrition have many essential properties, being some of the best sources of vitamins A, B12, D, bioactive compounds, and numerous minerals (calcium, iron, iodine, zinc, selenium, phosphorus, etc.), with high bioavailability (3, 4). Aquatic foods are also significantly different in their composition and properties from most terrestrial foods and are substantial for many coastal communities worldwide. These considerations have brought to the inclusion of the following three key messages in the published UN policy brief: Encourage diversified consumption of aquatic foods, including low-trophic aquatic foods; Ensure equitable and sustainable supply and production of aquatic foods; Democratize knowledge, data, and technologies. The following Research Topic of scientific publications contributes novel knowledge promoting Sustainable Fisheries, increasing the economic benefits from sustainable use of marine resources, and increasing research and technology for ocean health. It is organized around two main knowledge areas - Aquatic foods as marine resource for human nutrition, and Nutrition in aquaculture.

Ebrahimi et al. reviewed the methods for achieving synergistic and additive effects of natural marine bioactive compounds and extract combinations with anti-obesity, anti-inflammatory, antioxidant, and chemo preventive activities in the last two decades. While most marine bioactive combinations this far have been concentrated on potential synergies of fish oil and carotenoids, strategies to increase the number and diversity of marine bioactive combinations and develop marine-based functional foods with higher efficacy for disease prevention, are needed.

Zeng et al. examined the relationship between n-3 PUFA poor/rich seafood consumption and gout, the most prevalent inflammatory arthritis, by use of data from the National Health and Nutrition Examination Survey (NHANES) for US adults. A dose-response analysis showed, in the female group, non-linear relationship between n-3 PUFA rich seafood intake and the risk of gout. Additionally, the study provided proof-of-concept regarding the prevention potential for n-3 PUFA rich seafood against the harmful effects of purines in gout.

The integrated approach of the Samaki Salama project intervention (Blackmore et al.) aimed to promote sustainable fishing practices and improve the nutritional status of young children < 5 years of age in small-scale fishermen households in Kenya. The study provides an example of how to leverage multiple disciplines to address key challenges to human and environmental health and illustrates a pathway for scaling study innovations to other small-scale fisheries systems.

Sustainability in consumption has also been explored by Kendler et al. by studying the potential use of several underutilized marine species, namely flounder (*Platichthys flesus*), lemon sole (*Microstomus kitt*), megrim (*Lepidorhombus whiffiagonis*), plaice (*Pleuronectes platessa*), and thornback ray (*Raja clavate*), often captured as by-catch in Norway, as an opportunity to ensure future supply of high quality, sufficient fish for consumption by the ever-growing population. All five species showed remarkable nutritional quality in the distribution of digestible indispensable amino acid ratio (DIAA) and the two main n3 fatty acids - EPA and DHA.

One of the main challenges in aquaculture nutrition nowadays is the replacement of fish oil (FO) in aquafeeds, which implies identifying sustainable alternative sources of EPA and DHA, to ensure the naturally required LC-PUFA levels in marine fish.

Marques et al. evaluated different combinations of lipid sources rich in n-3 LC-PUFA, available on the market as a marine alternative to replace traditional fish oil (sardine oil), in diets for European sea bass (*Dicentrarchus labrax*). The analysis demonstrated that n-3 LC-PUFA rich sources from salmon oil, algae oil and a blend of micro and macroalgae (Algaessence Feed™) were viable solutions for the direct replacement of traditional fish oil or for preparation of combinations to fortify European sea bass.

In parallel, Mota et al. evaluated the potential of a commercial algae blend, composed of macroalgae (*Ulva* sp. and *Gracilaria gracilis*) and microalgae (*Chlorella vulgaris* and *Nannochloropsis oceanica*), in a plant-based diet (up to 6% on dry matter basis) upon the digestibility, gut integrity, nutrient utilization, growth performance, and muscle nutritional value of European seabass juveniles. The study demonstrated the beneficial effects of the commercial algae blend supplementation; however, feeding trials up to commercial-size fish are needed to fully assess its potential as feed.

The study conducted by Zhang et al. investigates by molecular approaches the effects of phenylalanine supplementation on gene regulation of growth, digestive capacity, antioxidant capability, and intestinal health of triploid rainbow trout (*Oncorhynchus mykiss*), fed with low fish meal diet.

Nguyen et al. examined the impact of different levels of dietary methionine concentrations, combined with elevated sea temperature on feed intake, brain expression of selected neuropeptides and melanocortin 4 receptor involved in appetite control in juvenile cobia (*Rachycentron canadum*). The study highlighted a significant effect of temperature and dietary methionine on appetite-related neuropeptide expression in juvenile cobia brains.

Guo et al. evaluated the potential use of Perilla, fish, or soybean oil as lipid sources in the diet of the Chinese giant salamander (*Andrias davidianus*), to fulfill the need for compound feed development. The study showed that all analyzed oils (Perilla, fish, and soybean) positively affect growth performance and specifically emphasized that Perilla oil, as fat source of *A. davidianus* compound feed, can enhance muscle quality and antioxidant capacity, boost immunity, promote lipid metabolism, and maintain liver and intestinal health.

In summary, the results from the above-mentioned studies and reviews represent a substantial knowledge contribution to: (1) establishing possibilities for increased sustainable future aquatic food consumption, e.g., through the development of novel functional foods and bioactives/ nutraceuticals with inclusion of alternative marine sources; (2), developing approaches for improved quality of cultured fish or alternative aquatic animal species, by use of novel feed sources and balanced feed diets. The integrated approach of the Samaki Salama intervention (Blackmore et al.) provides an example of how to leverage multiple disciplines to address key challenges to human and environmental health and illustrates a pathway for scaling study innovations to sustainability estimates of various small-scale fishery systems.

## Author contributions

MA: Writing – original draft, Writing – review & editing. LK: Writing – original draft, Writing – review & editing.
